# Intracardiac electrogram analysis may allow for prediction of lesion transmurality after pulsed field ablation of atria in a porcine model

**DOI:** 10.1016/j.hroo.2024.11.025

**Published:** 2024-12-05

**Authors:** Jernej Štublar, Tomaž Jarm, Lars Mattison, Bryan D. Martin, Megan Schmidt, Matevž Jan, Atul Verma, Paul A. Iaizzo, Daniel C. Sigg, Damijan Miklavčič

**Affiliations:** 1 Faculty of Electrical Engineering, University of Ljubljana, Ljubljana, Slovenia; 2 Cardiovascular Surgery Department, University Clinical Centre, Ljubljana, Slovenia; 3 Medtronic, Minneapolis, Minnesota; 4 Division of Cardiology, Health Centre, McGill University, Montreal, Canada; 5 Visible Heart Laboratories, Institute for Engineering in Medicine, University of Minnesota, Minneapolis, Minnesota

**Keywords:** Pulsed field ablation, Atria porcine model, Intracardiac electrograms, Bipolar vs unipolar iEGMs, Frequency analysis, Discrete wavelet transform, Current of injury, Lesion transmurality

## Abstract

**Background:**

Pulsed field ablation (PFA) is a novel cardiac ablation modality with an increasing clinical acceptance in treatment of atrial fibrillation due to its clinical efficacy and excellent safety profile. However, intraprocedural guidance for PFA to ensure durable pulmonary vein isolation (PVI) is lacking.

**Objective:**

We quantified changes in intracardiac electrograms (iEGMs) following PFA and radiofrequency ablation (RFA) and investigated their applicability for prediction of lesion transmurality.

**Methods:**

We induced 38 atrial lesions using PFA or RFA in 5 swine and monitored iEGMs continuously for up to 30 minutes postablation. The most characteristic changes in iEGMs were quantified after the decomposition using discrete wavelet transform, which allowed us to analyze the effects in separate frequency bandwidths.

**Results:**

After the ablation, we observed a reduction of bipolar iEGM amplitude (for PFA and RFA) and an increase in unipolar iEGM amplitude (predominantly for PFA). These changes were due to 2 mechanisms with different frequency content. The low-frequency content of unipolar iEGMs (1–16 Hz) further enabled us to discriminate between transmural and nontransmural lesions in the case of PFA. The rate of reduction of initially increased current-of-injury effect reflected in the low-frequency content of unipolar iEGMs within the first few minutes postablation was significantly higher and more pronounced for nontransmural lesions.

**Conclusion:**

This study shows that unipolar iEGMs can be used to differentiate between transmural and nontransmural atrial lesions within minutes after PFA in a porcine model, with implications for development of intraprocedural guidance of PFA procedures.

## Graphical abstract

**Figure:**
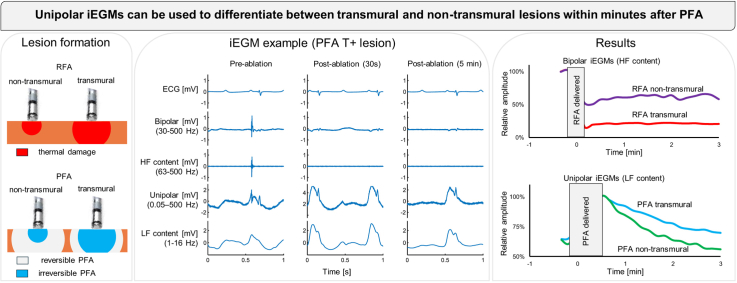

**Key Findings**

▪ Unipolar and bipolar intracardiac signals (iEGMs) differ minutes after pulsed field ablation (PFA) and radiofrequency ablation. The differences are larger in unipolar iEGMs due to appearance of a prominent current of injury (COI) after PFA.
▪ The characteristic changes in unipolar and bipolar iEGMs after ablation were quantified after signal decomposition into separate frequency sub-bands. The COI in unipolar iEGMs was mostly contained in the low-frequency sub-band between 1 and 16 Hz.
▪ The difference between transmural and nontransmural lesions after PFA was significant for the COI derived from unipolar iEGMs as soon as 1 minute postablation, which could be exploited for development of an intraprocedural guidance for lesion transmurality.

## Introduction

Pulsed field ablation (PFA) is an emerging, effective, cardiac ablation modality for treatment of atrial fibrillation based on electroporation.^1^, ^2^, ^3^, ^4^, ^5^ Electroporation is a phenomenon in which short high-voltage pulses transiently increase cell membrane permeability and consequently disrupt cell homeostasis.^6^ While the cells can regain their normal function after reversible electroporation, the term “irreversible electroporation” is used when membrane and cell damage ultimately leads to cell death.^7^, ^8^, ^9^ PFA offers increased safety in comparison with radiofrequency ablation (RFA) and cryoablation due to reduced collateral damage to the surrounding tissue that otherwise can result in esophageal fistula, phrenic nerve palsy, and pulmonary vein stenosis.^8^^,^^10^, ^11^, ^12^, ^13^ Furthermore, it is the unprecedented speed of applications and ease of achieving acute pulmonary vein isolations (PVIs) that is clinically attractive. This approach induces the immediate abolishment of bipolar intracardiac electrograms (iEGMs), which in part was responsible for the enthusiasm of early adopters of PFA technology as an alternative to thermal methods for cardiac ablation.

However, even with subtherapeutic PFA applications, the affected cardiac cells can transiently lose their abilities to generate and/or propagate action potentials, as evidenced by immediate disappearance of the sharp depolarization components in bipolar iEGMs. Therefore, the amplitude reductions and morphology changes of bipolar iEGMs are not a reliable predictive indicator of successful ablation even though they may still serve as a relative indicator of acute success of ablation.^3^ This leaves the operator essentially without any objective feedback during the procedure about the likelihood of a successful ablation to achieve a durable transmural lesion. Therefore, operators currently rely on the prescribed ablation protocols that are based on clinical and preclinical evidence to be successful in achieving PVI.

In the present study, we investigated bipolar and unipolar iEGM changes following PFA treatment in atria in comparison with RFA within a porcine animal model in an attempt to investigate the ability to distinguish between transmural and nontransmural lesions. The success of differentiating lesion transmurality would potentially provide us with intraprocedural guidance that we are currently lacking.

## Methods

All data are available in the main text or the Supplemental Materials. Raw data can be obtained from Medtronic pending a Material Transfer Agreement (contact the corresponding author, D.M.).

### Swine preparation and catheter placement

This preclinical study was conducted at the University of Minnesota Visible Heart Laboratories. The study protocol was approved by the Institutional Animal Care and Use Committee and conformed to the Guide for the Care and Use of Laboratory Animals.

A total of 5 swine were anesthetized using standard practices and maintained using isoflurane. Access was gained via the femoral and jugular veins. Where applicable, transeptal puncture was performed to gain access to the left atrium. A diagnostic catheter (MarinR CS; Medtronic) was placed through the jugular access into the superior vena cava, outside the cardiac silhouette on fluoroscopy, and was used as the unipolar iEGM reference.

Next, the ablation catheter (DiamondTemp; Medtronic) was introduced through the femoral sheath and unique ablation targets in the atria were chosen, namely the left or right atrial appendage, right atrial lateral wall, right pulmonary vein, superior vena cava, and tricuspid annulus. As is usually the case in swine hearts, only 1 right pulmonary vein was present in all 10 swine. The lesion locations were chosen based on tissue thickness (to have a mix of thin and thick tissue targets), rather than on clinical relevance of the location. The ablation catheter was placed in a targeted atria site and baseline iEGM recording was initiated. Then, either unipolar RFA or bipolar PFA (both proximal rings were used for current return for PFA) energy was delivered. PFA was delivered from a modified research PulseSelect generator (Medtronic), at voltages between 500 and 1500 V, and with 1–4 trains of pulses. RFA was delivered at doses between 50 W at 60 °C for 5 seconds and 50 W at 60 °C for 10 seconds. For each modality, delivered energies were titrated in attempts to achieve balanced numbers of transmural and nontransmural lesions. For PFA, all nontransmural lesions were created with the lowest dose (500 V, 1 train). Subsequently, the ablation catheter (serving also as the iEGM recording catheter) was left in place for up to 30 minutes of continued data collection after a given ablation. The catheter was then repositioned either within the left or right atrium and the protocol was repeated at the next location. In total, up to 6 atrial lesions were created per animal with PFA and/or RFA energies without overlapping the lesion sites.

### iEGM acquisition and analyses

iEGMs were acquired and digitized using a clinical recording system, using a 1 kHz sampling frequency (CardioLab; GE Healthcare). In addition to standard bipolar iEGMs with typical clinical filter settings (bandpass 30–500 Hz), unipolar iEGMs with a broadened bandpass filter setting (0.05–500 Hz) were recorded together with standard 3-limb electrocardiography (ECG). At each targeted site, the iEGMs were recorded for at least 20 seconds prior to PFA or RFA delivery, to establish baseline pretreatment values, and continued without interruption for either 10 or 30 minutes after the ablation.

The ECG recordings were visually examined, and a lead best suited for automatic QRS complex detection was selected. The So-Chan QRS detection algorithm was implemented in MATLAB R2021b (The MathWorks).^14^ To improve QRS detection, the chosen ECG signal was prefiltered using a high-pass filter set at 10 Hz, and the first 50 samples were set to zero to avoid the cases in which the signal started on a QRS complex, which would otherwise prevent the So-Chan algorithm from detecting the next valid QRS complex in the sequence. To isolate the interval of predominantly atrial electrical activity within each heartbeat, we selected a window of 150 ms duration starting 200 ms before the detected QRS peak. Parameters of iEGMs pertaining to changes induced by ablation were determined within such 150-ms intervals (see also Supplemental Figure 1).

iEGMs were processed and analyzed in MATLAB. The recordings were divided into nonoverlapping 10-second segments. Signals were inspected for abnormalities, typically caused by the amplifier saturation, poor electrical contact, loss of current return pathway, or detrimental movement artifacts. Segments with more than 20% of the heartbeats contaminated with such artefacts were excluded from the analysis. In the case of PFA (but not RFA), the energy delivery always induced a temporary saturation of signal amplifiers, and it took up to 30 seconds after PFA for the unipolar signals to become valid (ie, unsaturated) again. For this reason, the postablation values of unipolar iEGMs for both PFA and RFA were analyzed from 30 seconds postablation onward.

On each nonoverlapping 10-second segment, a discrete wavelet transform (DWT) was performed using the Daubechies order 6 orthonormal wavelet family as suggested by Morellato and colleagues.^15^ By using DWT (see the Supplement for details), iEGMs were decomposed into nonoverlapping frequency sub-bands, allowing separate observation and analysis of high-frequency (HF) and low-frequency (LF) ranges. Frequency range of 63–500 Hz was chosen empirically, as it captured most of the postablation changes in the HF range. The frequency range of 1–16 Hz, on the other hand, was chosen, as it captured most of the postablation changes in the LF range. In the Results, we show that the decrease of the peak-to-peak values in the HF range mostly reflected the disappearance of the depolarization component in bipolar iEGMs, while peak-to-peak values in the LF range mostly reflected the appearance or increase of the current-of-injury (COI) component after application of the ablation in unipolar iEGMs.

### Determining lesion transmurality

All lesions were allowed to mature for a minimum of 2 hours following the last energy application. Subsequently, swine were euthanized by administration of high-potassium cardioplegia directly to the aortic root and perfused to the coronaries while under anesthesia. Each lesion was identified by submerging the cardiac tissue in warm (37 °C) 1% triphenyl tetrazolium chloride (TTC) solution. Once lesions were identified, they were cross-sectioned and stained again in TTC to assess transmurality. To be considered transmural, the endocardial lesion needed to be continuous to the epicardium, as visualized using the TTC staining procedure.

### Statistical analyses

All signals were modeled using a linear mixture model approach. Linear mixed-effect models were utilized to account for repeated measures on an animal and estimate the difference in iEGMs between groups (transmural and nontransmural lesions), both overall average and the slope of change (postablation) over time. The iEGMs were modeled as a function of time after ablation, group, and swine identifiers. Time, group, and an interaction between time and group were treated as fixed effects, while swine identifier was treated as a random effect. An analysis of variance (ANOVA) was conducted to evaluate the significance of the time and time-by-group interaction fixed effects. Additionally, the significance of the difference between the 2 groups at each time point was assessed using either t test or Mann-Whitney test (after testing for normality and equality of the variance) as applicable. All tests were conducted using the R statistical software version 4.3.1 (R Foundation for Statistical Computing).

## Results

### Bipolar vs unipolar iEGM recordings

**Figure 1:**
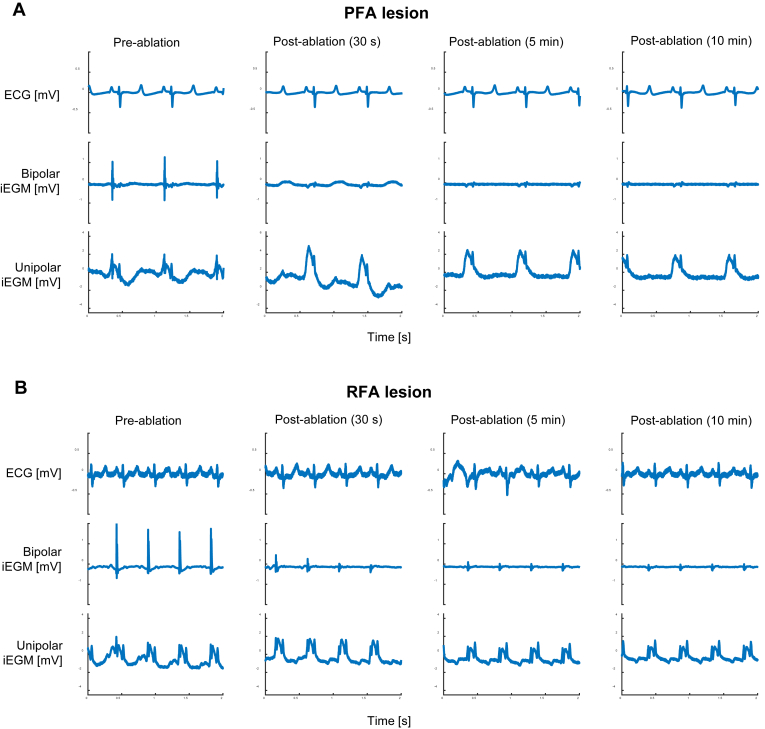
Raw unprocessed signals at 4 different time points relative to the energy delivery. Electrocardiography, bipolar intracardiac electrogram (iEGM) (bandpass 30–500 Hz) and unipolar iEGM (bandpass 0.05–500 Hz) signals are shown in the upper, middle, and bottom rows, respectively. A: Pulsed field ablation (PFA) lesion (1500 V, 4 pulse trains). B: Radiofrequency ablation (RFA) lesion (50 W at 60 °C for 10 seconds).

Both PFA and RFA energies induced profound changes in iEGMs immediately following the delivery of ablation energy, as shown in Figure 1. As expected, a decrease in bipolar iEGM amplitudes (mostly due to the near disappearance of the fast, sharp, and transient depolarization component in bipolar iEGMs) was observed for both energies. As reported previously,^16^^,^^17^ a pronounced increase in unipolar iEGM amplitudes (a slow, low-frequency phenomenon due to a large upward shift in the signal level following the atrial depolarization) after PFA was observed (Figure 1A). The changes in unipolar iEGMs resemble the COI phenomenon, observed for example at the site of implantation of a pacemaker lead,^18^ or recently described for the intramyocardial guidewire navigation technique.^19^ Both types of changes were seen to some extent for both treatment modalities (PFA and RFA), but the changes in unipolar iEGMs appeared more pronounced after PFA than RFA (compare the pre- and postablation unipolar signals in Figures 1A and 1B). The differences between them were further explored.

**Figure 2:**
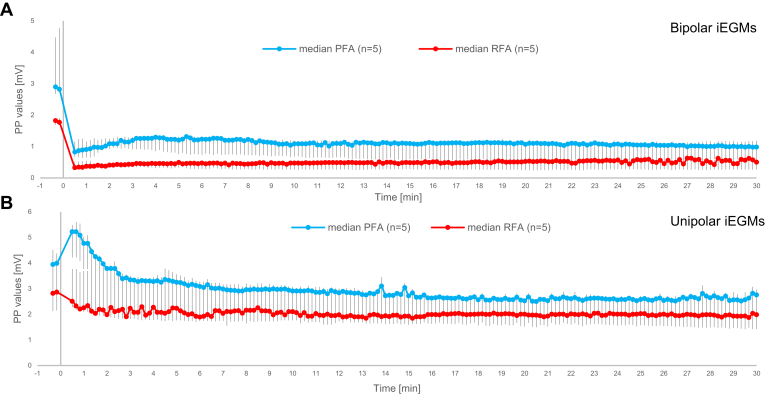
Comparison of changes in bipolar intracardiac electrograms (iEGMs) (A) and unipolar iEGMs (B) between pulsed field ablation (PFA) and radiofrequency ablation (RFA). The ablation energy was delivered at time 0. Absolute peak-to-peak (PP) values are shown (median and interquartile range for 5 lesion sites per treatment modality). Before averaging across the lesion sites, the individual values for each lesion for all time points (placed 10 seconds apart) were determined as the median values of PP values for all heartbeats within each 10-second interval centered at the chosen time point for the preablation period (the first 2 time points) and for the period of 30 minutes postablation.

We first determined the dynamics of postablation changes in iEGMs over time to find the most appropriate observation period to capture the most relevant changes. For this purpose, 5 treatment sites for each treatment modality were monitored continuously for 30 minutes postablation (the transmurality of lesions was not considered at this stage), as such an observation period was also recommended in 2017 in the expert consensus statement for radiofrequency catheter ablation of atrial fibrillation.^20^ These recordings served for the first comparison of changes between bipolar and unipolar iEGMs following PFA and RFA that are shown in Figures 2A and 2B, respectively. Peak-to-peak absolute values of unprocessed raw iEGMs are shown. Even though large differences of about 1 mV in baseline (preablation) median peak-to-peak values between the 2 groups were found, they were not statistically significant, and we believe that they were only coincidental and a consequence of a small sample sizes and large variability between lesion sites. Much smaller baseline differences in baseline values shown in the later figures (for larger sample sizes) support this hypothesis.

In bipolar iEGMs (Figure 2A), we observed a rapid reduction in overall peak-to-peak values immediately after the treatment for both PFA and RFA, with little to no subsequent recovery within 30 minutes following the treatment. On the contrary, in unipolar iEGMs (Figure 2B) PFA elicited a pronounced increase in peak-to-peak amplitudes 30 seconds after the applied treatment, followed by a relatively quick decrease (recovery) that appeared to be mostly completed within about 10 minutes postablation. In contrast to PFA, the initial increase after the applications of RFA was absent and most of the changes appeared to be finished within about 5 minutes post-treatment. In conclusion, because the most significant changes in all iEGMs appeared to be confined to the first 5–10 minutes, we limited the observation and signal recording in the continuation of our study to 10 minutes postablation.

### Frequency analysis of iEGM recordings

The observed morphological changes, that is, the attenuation (disappearance) of the sharp transient depolarization component (most notably in the bipolar iEGMs) and the appearance of the slow and much longer-lived manifestation of the COI phenomenon (in the unipolar iEGMs) led us to quantification of both types of changes induced by the treatments in different frequency ranges: in the HF range for the bipolar iEGMs and in the LF range for the unipolar iEGMs. Multiresolution decomposition using DWT was used for this purpose (see the Supplement for details).

**Figure 3:**
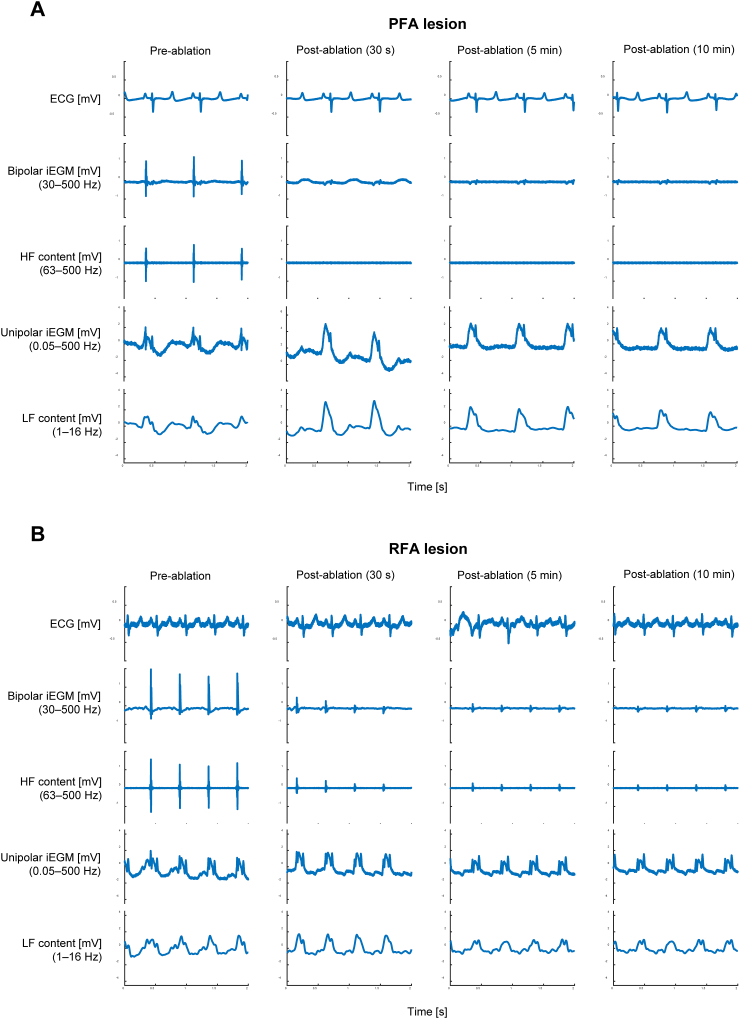
Isolation of the high-frequency (HF) and low-frequency (LF) content from bipolar and unipolar intracardiac electrograms (iEGMs), respectively, following the discrete wavelet transform (DWT) decomposition of iEGM at 4 different time points relative to the energy delivery. A: Pulsed field ablation (PFA) lesion; B: radiofrequency ablation (RFA) lesion. HF content: 63–500 Hz of bipolar iEGM. LF content: 1–16 Hz of unipolar iEGM. The same signals as in Figure 1 are shown. ECG = electrocardiography.

The result of DWT decomposition of iEGMs for the 2 lesion sites presented in Figure 1 is illustrated in Figure 3. Raw ECG and bipolar and unipolar iEGMs are shown in addition to the HF and LF contents extracted from the bipolar and unipolar iEGMs, respectively. The HF and LF contents were reconstructed from the empirically determined frequency sub-bands after the DWT decomposition in such a way that they captured the most of information belonging to the depolarization component of the bipolar iEGMs and to the COI component of the unipolar iEGMs, respectively. The entire DWT decomposition tree with all resulting frequency sub-bands is illustrated in Supplemental Figure 1. It is clear from both sets of signals in Figure 3 that the application of ablation energy had little to no effect on ECG signals.

In bipolar iEGMs, most of the energy of the signal corresponding to the sharp, transient depolarization component (best seen in row 2, column 1 in both panels of Figure 3) is contained in the frequency range of 63–500 Hz (for the HF content, compare signals in rows 3 and 2 of both panels of Figure 3). Figure 3 demonstrates how HF content practically disappeared immediately after the ablation (row 3 of both panels) with very little to no recovery within the first 10 minutes postablation. The amount and dynamics of this recovery varied between lesion sites and treatment energies.

In unipolar iEGMs (row 4 in both panels of Figure 3), most of the energy of the signal corresponding to the COI phenomenon after ablation (best seen in column 2) was contained in the frequency range of 1–16 Hz (for the LF content, compare rows 4 and 5 in both panels of Figure 3). The increase in COI was consistently observed immediately after ablation; however, this increase was more prominent after PFA. After the initial increase, the amount of COI gradually diminished within the first 10 minutes postablation. This observation suggested that the main differences in response between the lesions were due to different treatment energies (PFA or RFA). This hypothesis was further explored as follows.

### Differences in response between treatment modalities

**Figure 4:**
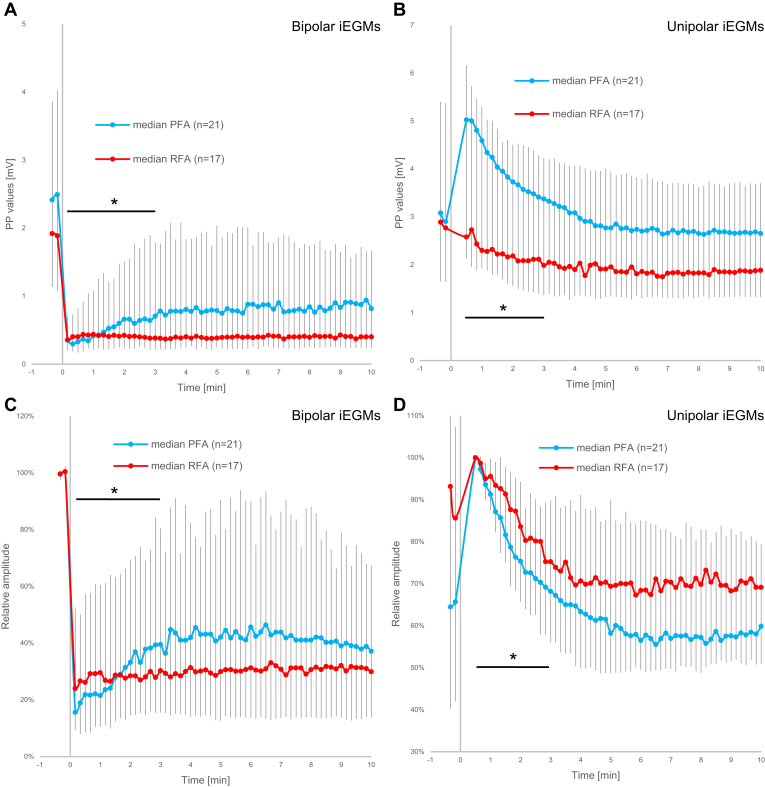
Comparison of changes in high-frequency (HF) content (bandpass: 63–500 Hz) of bipolar intracardiac electrograms (iEGMs) (A, C) and low-frequency (LF) content (bandpass: 1–16 Hz) of unipolar iEGMs (B, D) between pulsed field ablation (PFA) and radiofrequency ablation (RFA). The ablation energy was delivered at time 0. Absolute peak-to-peak (PP) values are shown (median and interquartile range) in panels A and B, representing the median values for the heartbeats within 10-second intervals for the preablation period (the first 2 data points) and for a period of 10 minutes after ablation. C: Data from panel A normalized to the first preablation value for each individual lesion before averaging. D: Data from panel B normalized to the first valid postablation value (30 seconds postablation) for each individual lesion before averaging. ∗*P* < .001, linear mixed-effect models—analysis of variance (time-by-group interaction) for the difference between the slopes on the interval between 30 seconds and 3 minutes postablation.

Differences in response to ablation between PFA and RFA ablation modalities, as quantified from bipolar iEGMs and unipolar iEGMs after DWT decomposition, are shown in Figure 4. The HF content of bipolar iEGMs and the LF content of unipolar iEGMs are shown before normalization (ie, absolute values [Figures 4A and 4B]) and after normalization (ie, relative values [Figures 4C and 4D]). In this comparison, all lesions for each treatment modality are still pooled together, regardless of the transmurality status of the lesions that subsequently developed at treatment sites (n = 21 for PFA and n = 17 for RFA).

Both PFA and RFA resulted in a pronounced reduction of the absolute peak-to-peak values of the HF content of bipolar iEGMs immediately after ablation (Figure 4A). For the RFA group, we observed no recovery after the initial drop within the first 10 minutes. However for the PFA group, the recovery was significant within the 3 minutes postablation (*P* < .001, linear mixed-effect models—ANOVA [time variable]). Furthermore, the difference in the slope of recovery between both groups within the 3 minutes postablation was statistically significant (*P* < .001, linear mixed-effect models—ANOVA [time-by-group interaction]).

We also observed a difference in response to ablation of the absolute peak-to-peak values of the LF content of unipolar iEGMs between PFA and RFA (Figure 4B). The difference between the 2 treatment modalities was statistically significant within the first 3 minutes postablation (*P* < .001, linear mixed-effect models—ANOVA [time-by-group interaction]). In case of PFA, the LF content (the COI phenomenon) reached the maximum level 30 seconds postablation—this also mostly coincided with the first readings after the amplifier saturation due to high-voltage pulse deliveries for PFA. This initial 73% increase was followed by the recovery phase, reaching the pretreatment value approximately 4 minutes after the ablation. In contrast, the initial postablation increase (if any) in the LF contents was absent after RFA. The final values of the LF content (10 minutes postablation) were lower than preablation. We touch on this surprising and counterintuitive observation in the Discussion.

Figures 4C and 4D present the same data as Figures 4A and 4B but in normalized forms. In Figure 4C, the peak-to-peak data of individual lesions from Figure 4A were normalized to the preablation values as recommended by Koruth and colleagues^21^ to compensate for the significant baseline variability. Similarly, in Figure 4D, the data from Figure 4B were normalized, but in this case to the postablation value which was reached 30 seconds after ablation (the earliest postablation moment valid for all lesions). In Figure 4C (HF content of bipolar iEGM signals), we can see that while the initial rapid decrease to about 20% of the preablation value was similar for both PFA and RFA, the subsequent recovery was only present for PFA and became statistically significant within 3 minutes postablation (*P* < .001, linear mixed-effect models—ANOVA [time variable]). Difference in the slope of the recovery between the 2 treatment modalities was statistically significant for the HF content of the bipolar iEGMs and for the LF content of the unipolar iEGMs (*P* < .001, linear mixed-effect models—ANOVA [time-by-group interaction]) (Figures 4C and 4D, respectively).

### Differences in response between transmural and nontransmural lesions

Already during the initial design of the study, the aim was to create both nontransmural and transmural lesions in similar proportions with both treatments. After confirming that the changes induced by the 2 treatment modalities had different characteristics, we looked at the differences in changes in iEGMs with respect to the final treatment outcome (ie, the transmurality and nontransmurality of lesions). We did this separately for each treatment modality (PFA and RFA) because one of the primary objectives of this study was to investigate for PFA in particular if it would be possible to detect the clinical outcome in terms of transmurality of the lesion based on the changes in iEGMs recorded immediately (within few minutes) after the ablation (ie, during the treatment procedure).

**Figure 5:**
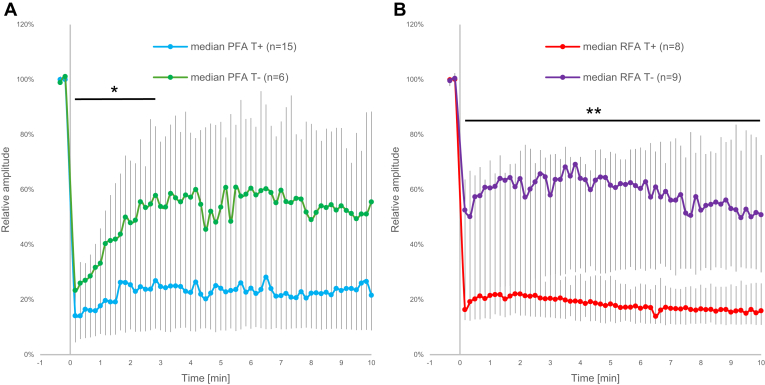
Comparison of changes in high-frequency content (bandpass: 63–500 Hz) of bipolar intracardiac electrograms with respect to the lesion status. The ablation energy was delivered at time 0. A: Pulsed field ablation (PFA); B: radiofrequency ablation (RFA). Individual absolute peak-to-peak values were normalized to the last preablation value before averaging. Medians with interquartile ranges are shown, representing the median values for the heartbeats within 10-second intervals for the preablation period (the first 2 data points) and for a period of 10 minutes after ablation. ∗*P* < .001, linear mixed-effect models—analysis of variance (time-by-group interaction) for the difference between the slopes on the interval between 30 seconds and 3 minutes postablation. ∗∗*P* < .05, Mann-Whitney test. T+ = transmural lesions, T– = nontransmural lesions.

**Figure 6:**
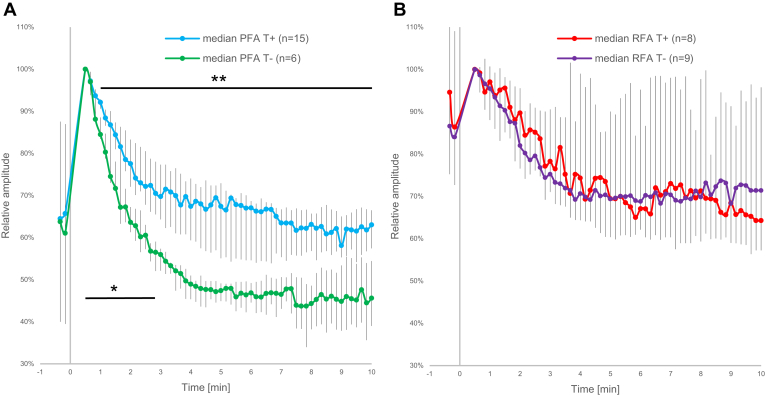
Comparison of changes in low-frequency content (bandpass: 1–16 Hz) of unipolar intracardiac electrograms with respect to the lesion status. The ablation energy was delivered at time 0. A: Pulsed field ablation (PFA); B: radiofrequency ablation (RFA). Individual absolute peak-to-peak values were normalized to the first valid postablation value (30 seconds postablation). Medians with interquartile ranges are shown, representing the median values for the heartbeats within 10-second intervals for the preablation period (the first 2 data points) and for a period of 10 minutes after ablation. ∗*P* < .001, linear mixed-effect models—analysis of variance (time-by-group interaction) for the difference between the slopes on the interval between 30 seconds and 3 minutes postablation. ∗∗*P* < .05, *t* test. T+ = transmural lesions, T– = nontransmural lesions.

For each treatment modality (PFA and RFA), the lesions were divided into transmural and nontransmural groups based on the postmortem gross pathological examination of the atrial tissue after staining with TTC, as described in the Methods. The differences between the two were evaluated separately for each treatment modality for the HF content of bipolar iEGMs (Figure 5) and for the LF content of unipolar iEGMs (Figure 6). Only normalized values are shown in Figure 5, Figure 6 (the same individual lesion data were used as in Figures 4C and 4D, respectively).

A sharp decline in the HF content (reflecting the disappearance of the depolarization component in the original bipolar iEGMs) was evident in both groups for PFA (Figure 5A). In the case of transmural lesions, the decrease to about 20% of the pretreatment value remained largely unchanged with very little indication of recovery within the first 10 minutes after the treatment. However, in case of nontransmural lesions, the initial decline (statistically indistinguishable from the transmural lesions) was followed immediately by a recovery phase, which plateaued at about 60% of the pretreatment value approximately 3.5 minutes after PFA. The slope of recovery between the 2 groups was statistically significantly different within the first 3 minutes postablation (*P* < .001, linear mixed-effect models—ANOVA [time-by-group interaction]).

The response observed in normalized HF data from bipolar iEGMs after RFA was similar to that after PFA in the sense that there was an initial rapid reduction after the ablation for both the transmural and the nontransmural lesions (Figure 5B). However, this reduction was clearly less pronounced for nontransmural lesions (to about 60% of pretreatment value) than in transmural lesions (to about 20% of pretreatment value). The difference between transmural and nontransmural lesions after RFA remained statistically significant (Mann-Whitney test, *P* < .05 for all time points) throughout the observed postablation period. Both the nontransmural and transmural groups showed no significant recovery after RFA (in contrast to the PFA nontransmural group).

When analyzing LF content of unipolar iEGMs after application of PFA, we observed a large initial increase in the signal, followed by a recovery phase for transmural and nontransmural lesions (Figure 6A). However, the recovery was faster and overall significantly more pronounced in case of nontransmural lesions. As early as 60 seconds postablation the difference between transmural and nontransmural lesions was already statistically significant (*t* test, *P* < .05). Furthermore, within the first 3 minutes postablation the 2 groups differed significantly also in the slope of recovery (*P* < .001, linear mixed-effect models—ANOVA [time-by-group interaction]).

In Figure 6B, the LF content of unipolar iEGMs for RFA groups is presented in normalized form. The initial increase in the LF content was much less pronounced than in the case of PFA. Furthermore, the recovery phase followed the same pattern for transmural and nontransmural lesions, with no significant differences between the two, a stark contrast to the results for PFA in Figure 6A.

## Discussion

Comparing bipolar and unipolar iEGMs following focal ablation in porcine atria using RFA and PFA energies allowed us to observe differences in dynamics and responses to different ablation energies. In addition, titrating PFA ablation energy to achieve transmural and nontransmural lesions allowed us to clearly distinguish between them. Specifically, in the present study we observed pronounced changes in signal morphology and the differential effects of ablation in porcine atria (especially in case of PFA) on peak-to-peak amplitudes of unipolar and bipolar iEGMs. As expected, in all cases the overall peak-to-peak amplitudes of bipolar iEGMs decreased after applications of PFA and RFA, while the overall peak-to-peak amplitudes of unipolar iEGMs increased consistently after application of PFA but to a lesser degree and less consistently after application of RFA. Similar observations have been recently reported by others,^16^^,^^17^^,^^21^ who investigated the irreversible and reversible iEGMs effects after PFA delivery. While Koruth and colleagues^21^ suggested pulsed field mapping for treatment of atrial tachycardias based on bipolar iEGMs, recovery dynamics, and reversible conduction block, others^16^^,^^17^ have quantified ST-segment elevation changes (a phenomenon that we prefer to call COI) in unipolar iEGMs in relation to observed lesions. Although different PFA systems were used, they all showed that iEGMs recovered in regions with reversible effect within 5 minutes, which is in agreement with our observations (see Figures 5A and 6A showing different rate of recovery between transmural and nontransmural groups within 3 minutes).

The term “ST-segment elevation” is perhaps more suitable when describing changes in ventricular iEGMs, but for observed changes in atrial iEGMs, as in our study, we believe that the term “COI” is more appropriate, as it is well established for both atrial and ventricular iEGM changes during pacemaker lead implantations.^18^ Amorós-Figueras and colleagues^16^ proposed that observed ST-segment elevations could be due to potassium releases into extracellular medium, changing transmembrane potential differences caused by reversible or irreversible electroporation. But it could also be a more functional phenomenon, as Bruce and colleagues^19^ were able to recreate observed ST-segment elevations in a simplified computational modeling only by making tissue adjacent to the electrode unexcitable. Electroporation is known to transiently increase cell membrane permeability and alter cellular homeostasis, making affected cardiomyocytes transiently nonexcitable.^22^, ^23^, ^24^, ^25^ In addition, it has been reported that high-voltage electric pulses can disrupt functioning of transmembrane channels.^5^ It has also been observed that gap junctions are affected by applying high-voltage pulses.^26^ Furthermore, electroporation has been described to cause the microcirculatory blood flow to shut down temporarily, which in turn results in local ischemia.^27^, ^28^, ^29^ Each of these effects known in electroporation literature alone and in combination may contribute to disrupted action potential propagation (ie, silencing of iEGMs). Finally, as electroporation renders membranes leaky/with increased membrane conductivity, it depolarizes the cells, rendering them (transiently) nonexcitable.^30^, ^31^, ^32^ These changes can be considered as tissue injury corresponding to phenomenon observed when the implantable cardioverter-defibrillator lead is fixed in the myocardium, causing electrical current flowing from injured/electroporated area toward healthy myocardium; thus, the proposed term, COI, can universally describe the observed phenomenon regardless of the anatomical location in the heart.^19^

For most lesions, the relative atrial iEGM values or LF content from unipolar iEGMs were smaller at the end of the observation period than before the treatment (Figure 6). This is a counterintuitive observation at first glance because it indicates that the COI was smaller 10 minutes after PFA delivery than before the treatment. This can be explained by generally large (and very variable) pretreatment LF content values observed for most lesion sites. Namely, because the LF content of unipolar iEGMs reflects the COI, we believe that the pretreatment values indicate the initial response of the tissue to the mechanical stress/injury caused by the contact between the catheter and the tissue (an effect that seems to gradually dissipate with time) in case of a stable contact. But as recently described, even subtle movements of measuring electrode inside the myocardium cause new acute COI signals.^19^ Consequently, the subsequent effect of ablation (the large increase of COI followed by the recovery phase as seen for PFA in Figures 4B, 4D, and 6A) is superimposed on the initial COI, and the two cannot be easily separated. A series of control measurements with the ablation catheter in place but without delivery of the ablation energy seem to support this hypothesis (see Supplemental Figure 2)—they show only the initially elevated COI (with large variability between sites) and the subsequent dissipation of the effect, the rate of which (again) can vary substantially between the sites.

A PFA index (in analogy to the RFA ablation index) was recently proposed. It is based on contact force and PFA dosing information,^33^ employing a similar approach as was reported to be efficient in the CLOSE protocol for RFA procedures, which is currently a gold standard for PVI.^34^ An analogous PFA index was recently tested in Dual energy for pulmonary vein isolation using dual-energy focal ablation technology integrated with a three-dimensional mapping system: SmartfIRE 3-month results (SmartfIRE study).^35^ PFA was used at posterior and inferior aspects of both PVI circles, due to proximity of esophagus, but 3 months’ remapping data showed that the vast majority (9 out of 12 patients) had reconnection at the posterior carina of the right circle, making PFA suboptimal in these regions. The ability to predict the lesion’s transmurality as early as 60 seconds after PFA application from analyzing iEGMs dynamics, as shown in our preclinical study, could perhaps serve as the basis for development of a PFA index applicable to all cardiac PFA systems.

Interestingly, already in 2010 Otomo and colleagues^36^ showed that morphological changes in unipolar iEGMs recorded during RFA had a 100% predictive value for creating transmural lesions. This hypothesis was tested in a clinical study^37^ but was never fully adopted in clinical practice.^38^, ^39^, ^40^ In our study, we did not find a significant difference between transmural and nontransmural lesions in unipolar signals following RFA (Figure 6B). In contrast, the unipolar iEGM changes appeared to have potentially predictive value for the transmurality of lesions after PFA (Figure 6A).

### Limitations

The observed findings cannot be directly translated into humans, as this study was conducted in healthy porcine hearts in vivo. Hence, further studies are needed to first corroborate our findings and to demonstrate clinical applicability.

We used a PFA research generator with the waveform intended for PVI using a multipolar loop catheter (PulseSelect). The waveform was not optimized for the solid tip catheter used in this study (DiamondTemp). To achieve nontransmural lesions, a subclinical PFA dose of 500 V and 1 train was used. However, even at this lowest dose, 7 out of 13 lesions were identified as being transmural, which resulted in unbalanced sample sizes of transmural and nontransmural groups for PFA. On the other hand, RFA dose titrations enabled us to produce balanced sizes of transmural and nontransmural groups; the larger dose of RFA (10-second delivery) produced 6 out of 8 transmural lesions and the lower dose (5-second delivery) produced 7 out of 9 nontransmural lesions.

The solid tip ablation catheter (DiamondTemp) that was used did not have the capability to measure contact force, so the quality of contact and its stability were monitored only on x-ray film and via observation of initial EGM signal amplitudes: thus, the impact of contact force on PFA lesion creation could not be studied. Temperature regulation system capability was used for RFA delivery but not for PFA. Lesion sites with observed movement artifacts in the recorded signals were excluded from analysis (4 out of 42 applications).

### Conclusion

In the present preclinical study, different behavior of iEGMs after PFA compared with RFA was shown, which was most obvious for unipolar iEGMs. Even though the immediate disappearance of bipolar iEGMs occurred with PFA, this had less predictive value for the transmurality of the induced lesions than the recovery dynamics observed in unipolar iEGMs. The COI recovery rate observable in unipolar iEGMs within 1 minute after delivery of PFA can be used to predict lesion transmurality in a porcine model, which could be the basis for development of a universal and more reliable PFA index.

## Appendix Supplementary Data

Supplementary Figure 2

Supplementary docx
