# Supplementary figures and images for: Intracardiac electrogram analysis may allow for prediction of lesion transmurality after pulsed field ablation of atria in a porcine model
Source: Heart Rhythm O2. 2024 Dec 5;6(3):350–61. doi: 10.1016/j.hroo.2024.11.025 (PMC11973674; doi:10.1016/j.hroo.2024.11.025)

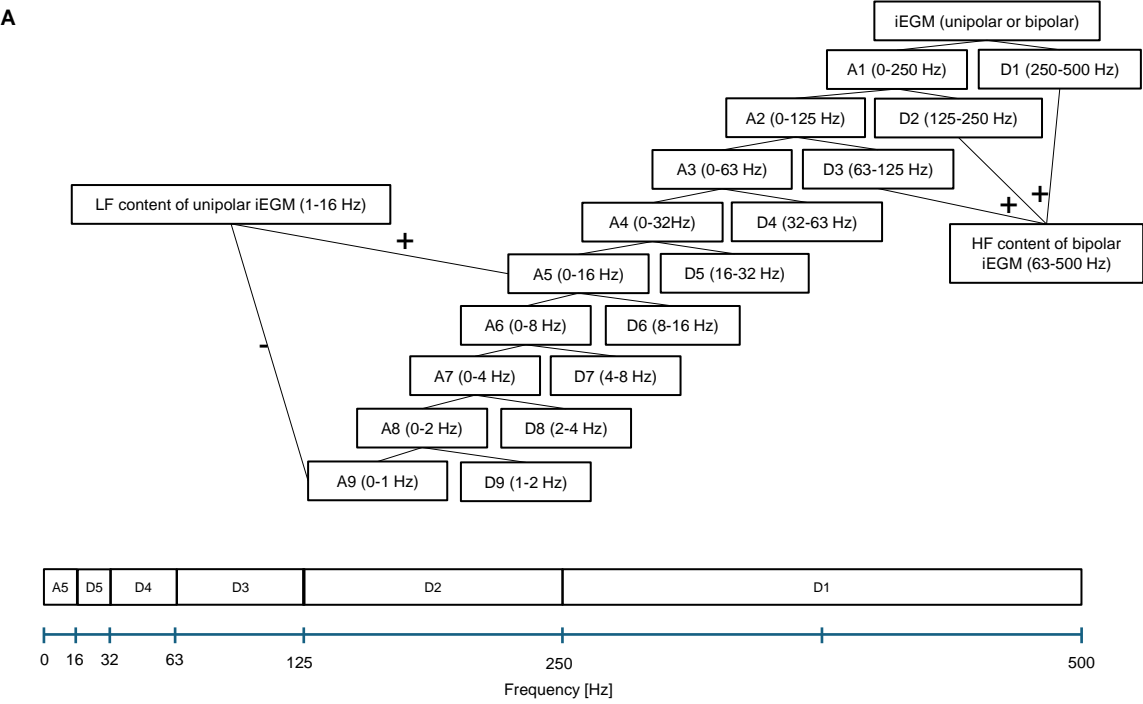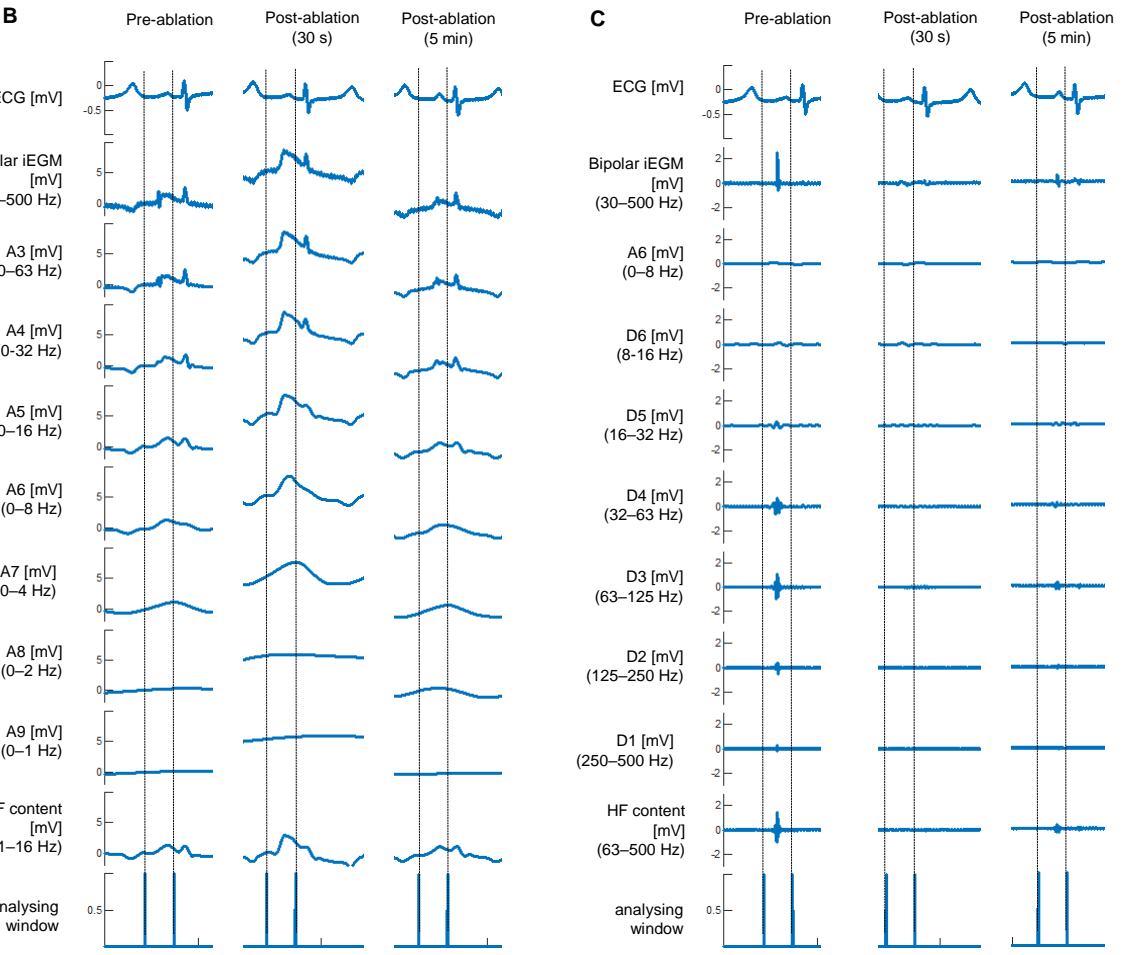

Supplement: Supplementary Figure 1 [file mmc1.pdf]

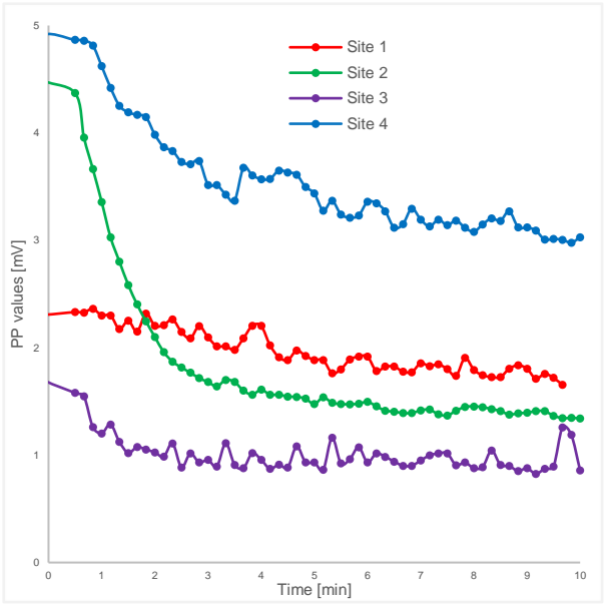

Supplement: Supplementary Figure 2 [file mmc2.pdf]
