# Supplementary material for: Intracardiac electrogram analysis may allow for prediction of lesion transmurality after pulsed field ablation of atria in a porcine model
Source: Heart Rhythm O2. 2024 Dec 5;6(3):350–61. doi: 10.1016/j.hroo.2024.11.025 (PMC11973674; doi:10.1016/j.hroo.2024.11.025)
Supplement: Supplementary docx [file mmc3.pdf]

Supplement

Morphological changes in intracardiac electrograms and discrete wavelets transform

After delivery of ablation energy we observed two profound and characteristic morphological changes in iEGMs (see also Figure 1): the reduction (or disappearance) of the highly localized and "sharp" high-frequency depolarization component (most prominently displayed in bipolar iEGMs) and the appearance (or large increase) of a slower and spread-out (spanning the interval between the local depolarization and the T wave) elevated "hump" in unipolar iEGMs (the low-frequency COI – Current-of-Injury phenomenon). To quantify these changes, we decomposed the iEGMs into separate frequency subbands that isolated these two distinct changes by using discrete wavelet transform (DWT) analysis. The theoretical and practical background of DWT can be found in numerous sources.[S1] Briefly, DWT-based multiresolution analysis is a method used to observe signal characteristics in frequency and time domains simultaneously and is particularly well-suited to capture transient components in nonstationary signals (specifically, the depolarization component and COI phenomenon in our study). Different "families" of wavelets with different properties can be used. Wavelets are test functions used for decomposition, analogous to the complex exponential function used in Fourier analysis. In our study we adopted the Daubechies order 6 (D6) orthonormal wavelet as suggested by Morellato et. al.[S2] who also used DWT for analysis of iEGM signals. We also tried some other wavelet families, but the results were very similar (not shown here), so at this stage we made no further attempt to optimize the choice of the wavelet family for this study.

The principle of DWT decomposition is illustrated in Supplement Figure 1A. The procedure starts with the original iEGM. DWT decomposition is performed by applying a special pair of low-pass and high-pass decomposition filters, specific to the chosen wavelet family, to the signal, followed by down sampling of both filtered versions of the signal. This results in two sets of DWT coefficients, the "approximation" of level 1 (A1) and the "detail" of level 1 (D1) coefficients, which essentially contain the original signal information in lower half and higher half frequency sub bands (0-250 Hz and 250-500 Hz in our case, as illustrated in Supplement Figure 1A). In DWT, applying this procedure recursively on each level of the decomposition tree splits the frequency range of the approximation of the previous level in half. A 9-level DWT was performed as shown in Supplement figure 1A. The complete information of the signal was thus split into a sequence of non-overlapping frequency subbands A9, D9, D8, D7,…, D1. We established empirically that the depolarization component in bipolar iEGMs (the HF content) was best described by the combination of components D3-D1. On the other hand, the COI phenomenon (the LF content) appeared to be captured best by component A5 from which the content of A9 was subtracted (in other words, the content of components D9-D6). To arrive at the HF and LF contents, the selected components were then combined in the process of DWT reconstruction, which iteratively applies a special pair of low-pass and high-pass reconstruction filters only to the combination of the selected components from the DWT decomposition tree. Supplement Figure 1B and 1C shows the relevant components for reconstruction of the LF and HF contents for unipolar and bipolar iEGM respectively for one single heartbeat. DWT analysis was performed in Matlab using the Wavelet Toolbox functions.[S3] This approach was also computationally undemanding enough that it could be implemented for real-time use in clinical systems.

Control measurements

We also conducted a series of control measurements (without delivery of ablation energy) to quantify and characterize the large initial baseline COI, which was observed in unipolar iEGM signals at ablation sites in the main part of the study. Originally, we intended to use this information to compensate for the highly variable COI observed at treatment sites that was not caused by the ablation. Supplement Figure 2 shows the evolution of LF content of unipolar iEGMs for four control measurement sites over a period of 10 minutes following the establishment of contact between the catheter tip and the atrial endocardial wall. These four individual traces (selected specifically to

illustrate the extent of variability) are directly comparable to averaged curves (medians for 21 and 17 lesion sites after PFA and RFA respectively) in Figure 4B.
Large initial COI variability can be observed in Supplement Figure 2 at time zero, spanning the range of 2 – 5 mV, which is like the interquartile ranges for baseline COI before ablation in Figure 4B. All curves in Supplement Figure 2 show clearly (and this was also the case for the rest of control measurements not shown here) that the COI gradually and monotonously declined over the period of 10 minutes. However, we found no apparent correlation between the rate of this decline (or "recovery") and the initial value, as the curves (Site 1-4) in Supplement Figure 2 demonstrate. This means that extracting the COI contribution induced only by the ablation from the total COI observed after the ablation would be practically impossible.

As the ablation catheter stability was only monitored by x-ray (the initial stability was established and rechecked at sporadic intervals, with the last check made at the end of the observation period), we can speculate that the very smooth and relatively rapid COI recovery (Supplement figure 2; site 2) means that the catheter to tissue contact at this particular site was relatively stable, while on the other hand slower recovery with intermittent fluctuations of COI (other three sites) could be due to slight catheter movements generating new acute COI elevations.[18] Use of a 3D mapping system and/or contact force sensing ablation catheters could mitigate this issue and make the positioning of the catheter more stable, the measurements less variable and potentially the control measurements useful for compensation for the "background" COI in measurements taken after the ablation.

Supplement Figure legends

Supplement Figure 1: DWT decomposition principle (A) and an example of reconstruction of the LF content from a unipolar iEGM (B) and the HF content from a bipolar iEGM (C) on the level of a single heartbeat, at three different time points relative to PFA delivery (before-ablation, 30 seconds post-ablation, 10 minutes post-ablation). A: DWT decomposition tree (9 levels) to separate the signal content into frequency subbands with indication of the components used to reconstruct the LF and HF contents from the unipolar and bipolar iEGM respectively. B: components of DWT decomposition relevant for reconstruction of the LF content from unipolar iEGM. ECG is shown for reference, dashed vertical lines indicate the time interval within which the signal was evaluated. C: the same as B, but for reconstruction of the HF content from bipolar iEGM.

Supplement Figure 2: Comparison of changes in LF (low-frequency) content (bandpass: 1 Hz – 16 Hz) of unipolar iEGM signals for four individual control measurement sites (sham lesions) over a period of 10 minutes after the catheter tip was brought into contact with the endocardial atrial wall. The same procedure was used as for PFA or RFA ablation sites, but in this case no energy was delivered. Absolute peak-to-peak values are shown representing the median values for the heartbeats within non-overlapping 10-second intervals.

References

S1. P.S. Addison, The Illustrated Wavelet Transform Handbook: Introductory Theory and Applications in Science, Engineering, Medicine and Finance, 2017, CRC Press; Boca Raton (FL).
S2. M. Haziq and K. Azman, So-Chan QRS detection algorithm, Available at https://www.mathworks.com/matlabcentral/fileexchange/61230-so-chan-qrs-detection-algorithm (Accessed November 2023).
S3. Matlab Wavelet Toolbox, Available at https://uk.mathworks.com/help/wavelet/index.html?s_tid=CRUX_lftnav (Accessed 4 October 2024).
